# Virtual reality simulation with eye-tracking feedback versus mannequin-based training for situational awareness in trauma management under simulated emergency department interruptions in Iran: a pilot randomized controlled trial

**DOI:** 10.3352/jeehp.2026.23.8

**Published:** 2026-04-29

**Authors:** Navaz Emadi, Rita Mojtahedzadeh, Seyyed Farshad Allameh, Kamal Basiri, Aeen Mohammadi

**Affiliations:** 1 Department of Medical Education, School of Medicine, Tehran University of Medical Sciences, Tehran, Iran; 2 Center of Excellence for E-learning in Medical Education, School of Medicine, Tehran University of Medical Sciences, Tehran, Iran; 3 Department of Internal Medicine, School of Medicine, Imam Khomeini Hospital Complex, Tehran University of Medical Sciences, Tehran, Iran; 4 Department of Emergency Medicine, Sina Hospital, School of Medicine, Tehran University of Medical Sciences, Tehran, Iran; Hallym University, Korea

**Keywords:** Advanced trauma life support care, Situational awareness, Eye-tracking technology, Simulation training, Virtual reality

## Abstract

**Purpose:**

To primarily examine the feasibility of implementing eye-tracking–based feedback within a virtual reality (VR) trauma simulation with realistically simulated emergency department interruptions, and to explore preliminary changes in situational awareness (SA) (primary outcome), Advanced Trauma Life Support (ATLS) performance, and trauma-management errors (exploratory outcomes) compared with conventional mannequin-based simulation.

**Methods:**

In this pilot randomized pretest–posttest study, 35 medical interns were assigned to VR training with eye-tracking heatmap feedback (n=17) or mannequin-based training with instructor verbal feedback (n=18). SA (modified Situation Awareness Global Assessment Technique), ATLS checklist performance, and trauma-management error scores were measured before and after the intervention. Within-group changes were tested with the Wilcoxon signed-rank test, and between-group differences were compared using the Mann-Whitney U test on change scores (Δ=post−pre), with effect sizes reported as r.

**Results:**

Baseline pretest performance did not differ significantly between the groups. Both groups improved in SA and ATLS performance (all P<0.001) and reduced error scores (VR: P=0.004; mannequin: P<0.001). In exploratory between-group comparisons, the VR group showed numerically greater improvements in SA (mean change 6.59 vs. 3.11; P=0.006, r=0.46), ATLS performance (22.12 vs. 8.22; P=0.003, r=0.48), and error reduction (–9.36 vs. –3.61; P=0.005, r=0.47). Given the pilot design, these differences should be interpreted as preliminary signals.

**Conclusion:**

In this pilot study, both training modalities were associated with improved SA and ATLS performance and with fewer errors, with point estimates favoring the VR condition. These preliminary signals suggest that VR with eye-tracking feedback may be a promising option for trauma training in interruption-rich, emergency department-like settings and warrants further evaluation in larger studies.

## Graphical abstract

[Fig f4-jeehp-23-08]

## Introduction

### Background/rationale

Clinical environments are inherently complex, requiring clinicians to synthesize multiple information streams to make rapid, high-stakes decisions, particularly in settings like the emergency department (ED) where interruptions are frequent and can undermine performance [1]. A critical cognitive skill supporting effective decision-making under such conditions is situational awareness (SA), defined as the perception of environmental elements, comprehension of their meaning, and projection of future states [2]. In EDs, a high burden of workflow interruptions has been directly associated with declines in physician SA [3].

Simulation-based training offers an effective pedagogical approach for cultivating SA by allowing deliberate practice in high-risk scenarios without compromising patient safety [4,5]. Recent technological advances have enabled the integration of virtual reality (VR) with eye-tracking, creating immersive environments in which trainees’ visual attention and scanning behaviors can be objectively captured and measured [6]. Eye-tracking technology provides a real-time window into cognitive processes that underpin SA by recording gaze distribution, fixation patterns, and dwell time on critical cues [7]. When these data are transformed into heatmaps and reviewed during debriefing, they can reveal missed cues, inefficient search strategies, and attentional traps that might otherwise go unnoticed in conventional feedback [6]. This capability is particularly relevant for interruption-rich ED settings, where the ability to rapidly detect and interpret critical cues directly affects clinical performance and patient safety. However, it remains uncertain whether eye-tracking-based feedback within an immersive VR environment can demonstrably enhance trainees' SA and clinical performance when training scenarios incorporate the realistically simulated interruptions characteristic of the ED [8]. Clarifying the potential added value of this approach, compared to conventional mannequin-based simulation, is essential to guide the design of interruption-sensitive trauma education and to inform future, more definitive evaluations [8].

### Objectives

This pilot study primarily assessed the feasibility of implementing eye-tracking–based feedback in a VR trauma simulation under simulated ED interruptions. Feasibility was defined pragmatically as the ability to recruit eligible interns and deliver the intervention within available logistical capacity. SA was designated as the primary outcome, while changes in ATLS performance and ATLS error scores were explored as secondary outcomes to inform a future adequately powered trial.

## Methods

### Ethics statement

The study was approved by the Ethics Committee of Tehran University of Medical Sciences (approval no., IR.TUMS.MEDICINE.REC.1402.058). Written informed consent was obtained from all participants before enrollment in the study.

### Study design

**Fig. 1.**
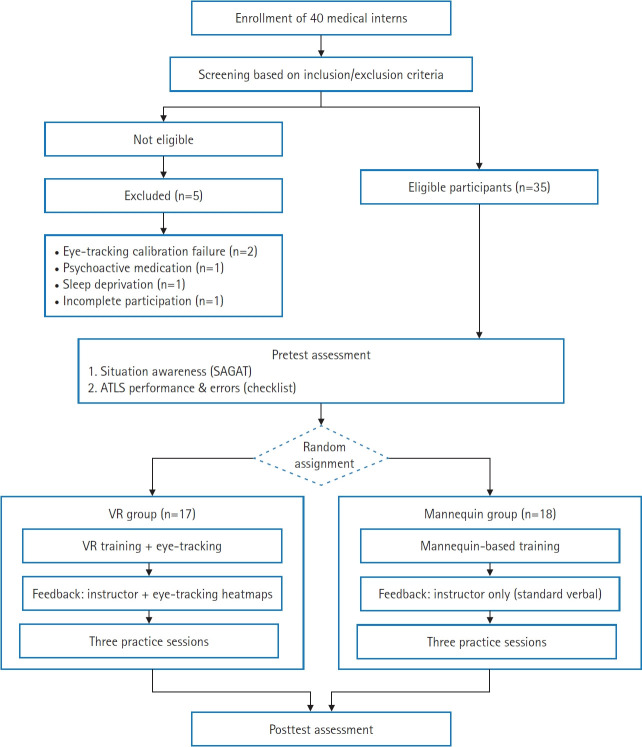
Flowchart of the educational intervention implementation. SAGAT, Situation Awareness Global Assessment Technique; ATLS, Advanced Trauma Life Support; VR, virtual reality.

A pilot randomized experimental pretest–posttest controlled design was used. The primary aim was feasibility, namely, to determine whether eye-tracking–based VR feedback could be implemented in this setting using the available pool of eligible interns and existing simulation resources. Analyses of SA, ATLS performance, and error scores were exploratory and intended to inform the design of a future adequately powered trial (Fig. 1).

### Setting

The study was conducted at the Clinical Skills Center of Tehran University of Medical Sciences, Iran, between August and November 2025.

### Participants

Medical interns were recruited through convenience sampling. In this pilot study, the sample size was pragmatically determined based on feasibility, including the number of eligible interns available during the study period and the logistical capacity for simulation delivery; therefore, no formal a priori power calculation was conducted. A total of 40 interns were initially enrolled, of whom 5 were excluded before randomization because of eye-tracking calibration failure (n=2), use of psychoactive medication (n=1), recent sleep deprivation (n=1), or incomplete participation (n=1). The remaining 35 participants were randomly assigned to either the VR training group (n=17) or the mannequin-based training group (n=18) using a computer-generated random sequence prepared by an investigator not involved in enrollment or assessment. Allocation concealment was ensured by using sequentially numbered, opaque, sealed envelopes opened after baseline assessment. Participant enrollment and group allocation were performed by separate independent researchers. Eligibility criteria included completion of at least 1.5 credits of the emergency medicine internship, a minimum of 6 months of internship experience, and at least 1 month of ED rotation. Exclusion criteria comprised visual or technical limitations affecting eye-tracking, use of psychoactive medications, recent severe sleep deprivation, or incomplete participation. All randomized participants were included in the final analysis.

### Intervention design

Two educational interventions were developed: a VR–based training program incorporating eye-tracking technology and a conventional mannequin-based training program, both aimed at teaching trauma management in a simulated ED environment with realistic interruptions.

#### Emergency interruptions identification and scenario development

To design an interruption-rich ED simulation, sources of interruption were first identified through an evidence review [9] and then refined by an expert panel (n=10; ≥5 years of ED experience) using the nominal group technique, which added ED overcrowding, lack of interprofessional cooperation, and concurrent educational activities. The prioritized interruptions were embedded into a high-acuity trauma scenario based on the 11th edition of ATLS, involving multiple simultaneous patients, and were iteratively revised and validated by the panel.

#### Development of educational content

An ATLS-based scenario was enacted by 21 trained personnel in a simulated ED and recorded as a 360° physician-perspective video, which was then converted into an interactive VR module using 3DVista (https://www.3dvista.com/en/) (Supplement 1). Custom Unreal Engine software (Epic Games) for the HTC VIVE Pro Eye (HTC Corp.) captured gaze trajectories and generated heatmaps, which were validated through multistage calibration and testing. The mannequin intervention retained the same scenario structure and instructional content, replacing standardized patients with a high-fidelity mannequin to ensure standardized, repeatable delivery.

### Intervention implementation

After pretesting (SA assessed using a modified Situation Awareness Global Assessment Technique [SAGAT] and ATLS performance/error scores measured using a researcher-made checklist), participants were allocated to the VR or mannequin group through simple randomization.

#### VR group intervention

**Fig. 2.**
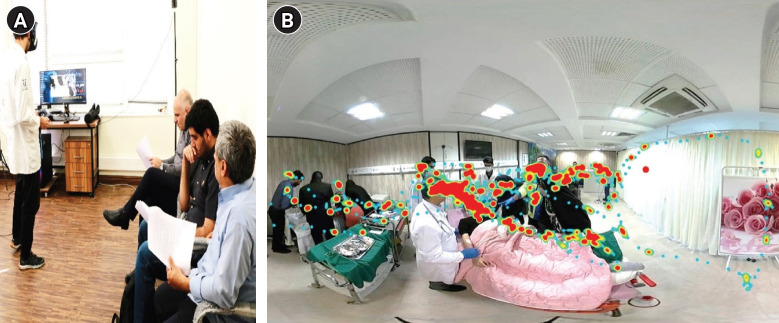
(A, B) show key elements of the virtual reality-based intervention: the eye-tracking heatmap feedback session (A) and an example of a generated eye-tracking heatmap (B). Written informed consent was obtained from all participants before enrollment in the study.

Before training, participants completed eye calibration and received standardized instructions on headset operation. Each participant then managed a multi-trauma patient with unstable vital signs in a simulated ED using an HTC VIVE Pro Eye headset, following ATLS protocols. Realistic interruptions (e.g., bystander interference, device alarms, background conversations, staff entry, and abrupt changes in patient condition) were embedded consistently across sessions. Each scenario lasted approximately 6 minutes and was immediately followed by instructor-led feedback. Eye-tracking heatmaps were used during debriefing to review attention to critical cues (e.g., the vital signs monitor and bleeding source) and to provide SA–focused feedback. Participants completed the session 3 times within 1 week and received personalized feedback after each repetition (Fig. 2).

#### Mannequin group intervention

Participants attended a brief orientation session covering the objectives and procedures. Each participant individually completed a 6-minute high-fidelity simulated ED scenario requiring assessment and management of a multi-trauma patient with unstable vital signs according to ATLS protocols. Scripted roles were performed by 18 trained individuals from the content development team, while ED ambient sounds (alarms, conversations, and background noise) were delivered via speakers. Performance was concurrently observed by 3 emergency medicine faculty members, followed by individualized feedback emphasizing decision-making under stress, SA, and management in the presence of disruptions. Participants completed the session 3 times within 1 week and received personalized feedback after each repetition. To minimize potential performance bias, several aspects of the intervention were standardized across both groups. Debriefing sessions in both the VR and mannequin groups were conducted by the same team of 3 emergency medicine faculty members with equivalent teaching experience. Each debriefing session lasted approximately 15–20 minutes, and the duration was kept comparable across both groups and all participants. The faculty-to-learner ratio was consistent for all sessions in both groups. Although cognitive load was not formally measured in this pilot study, efforts were made to maintain similar levels of task complexity and environmental stressors across both training modalities.

### Data collection

Data collection was conducted at 2 time points (pretest and posttest). SA was evaluated using a modified SAGAT, while ATLS performance and error scores were quantified using a researcher-developed checklist.

#### Pretest

SA was measured using a modified SAGAT during an 8-minute physically reconstructed simulated ED trauma scenario (14 minutes including freeze points), based on ATLS, featuring a critically unstable standardized patient (blood pressure [BP] 69/38 mm Hg, pulse rate [PR] 178 bpm, oxygen saturation [SpO_2_] 87%, respiratory rate [RR] 37) with moulage, distressed family members, and realistic environmental distractors and interruptions. Twenty-one trained role-players ensured standardized scenario delivery. A blinded independent assessor administered SAGAT at 3 predefined freeze points, beginning 3 minutes into the scenario, and evaluated ATLS performance and error scores using an ATLS-derived researcher-made checklist (11 multi-step items weighted by clinical importance) between freeze points. To minimize contamination, participants were quarantined until completion of all assessments.

#### Posttest

The posttest scenario differed from the pretest but was matched in difficulty and trauma severity; vital signs were comparable in severity, although not identical. To ensure equivalence between the pretest and posttest scenarios, both scenarios were independently evaluated by an expert panel of emergency medicine specialists involved in the study design. The experts rated the scenarios based on predefined criteria, including trauma severity, clinical decision complexity, number of critical cues, and interruption intensity, using a structured difficulty rating scale. Inter-rater agreement was reviewed, and consensus was reached indicating that the 2 scenarios were comparable in overall difficulty and educational demands. Environmental conditions and standardized realistic interruptions were kept consistent to facilitate outcome comparison. The posttest was also scored by an assessor blinded to group allocation, and participants were instructed not to disclose their intervention group to the assessor.

### Data collection instruments

#### SA

SA was assessed in this study using a modified version of the SAGAT. This instrument measures SA across 3 distinct levels: perception, comprehension, and projection [2]. To adapt SAGAT to the clinical scenario of ATLS intervention, the questionnaire was redesigned into a 17-item instrument, comprising 7 items for the perception level, 4 items for the comprehension level, and 6 items for the projection level (Supplement 2). All questions were scored dichotomously (true/false), with a total score ranging from 0 to 17. The SAGAT was administered at 3 predetermined time points, referred to as freeze points. These points were established based on content analysis of the ATLS scenario for the purpose of measuring the 3 components of SA (perception, comprehension, and projection), in accordance with protocols suggested in previous studies [2].

The first freeze point was activated at the third minute after the start of the scenario. The subsequent freeze points occurred sequentially at minutes 7 and 11 of the scenario. The duration of the freeze in each scenario execution was kept consistent. The active SAGAT scenario lasted approximately 8 minutes. However, the total assessment duration was 14 minutes because the simulation was paused at predefined freeze points for SAGAT administration. Therefore, the 3-, 7-, and 11-minute time points refer to cumulative elapsed time, including pauses for situation awareness assessment. The 17 SAGAT items were distributed across the 3 freeze points to capture different levels of SA: perception (7 items), comprehension (4 items), and projection (6 items), with items allocated according to the clinical progression of the scenario.

The face and content validity of the instrument were confirmed by 11 expert emergency medicine specialists, each with more than 3 years of clinical experience. To evaluate test–retest reliability, the questionnaire was re-administered after a 2-week interval to a sample comparable to the target population. This time interval was selected to minimize potential memory effects while maintaining relative stability in participants’ knowledge and experience. The results demonstrated strong correlations between the 2 administrations (r=0.95 for perception, r=1.00 for comprehension, and r=0.89 for projection), indicating high test–retest stability of the instrument. Furthermore, internal consistency was assessed using Cronbach’s alpha (α=0.77 in the first administration and α=0.79 in the second), showing acceptable homogeneity of the items in measuring the construct of SA.

#### ATLS performance and error assessment

ATLS performance and errors were evaluated using a researcher-developed, observation-based checklist derived from the 11th edition of ATLS (American College of Surgeons, July 2025) (Supplement 3). Interns managed a standardized multi-trauma simulation with unstable vital signs (BP 69/38 mm Hg, PR 178 bpm, SpO_2_ 87%, RR 37). The instrument comprised 11 multi-step items with conditional branching, and actions were scored using clinically weighted points (up to +5 for correct steps and −5 for unsafe or incorrect steps). Overall ATLS performance was calculated as the net score (sum of positive and negative points; range, −31 to +54). Errors were operationalized as an error (penalty) score, computed as the absolute value of the cumulative negative points, such that higher values indicated more frequent and/or more severe errors. Content and face validity were confirmed by 9 emergency medicine experts, internal consistency was acceptable (Cronbach’s alpha=0.713), and inter-rater reliability between 2 evaluators was good (weighted Cohen’s kappa=0.78).

### Data analysis

The data were analyzed using IBM SPSS ver. 26.0 (IBM Corp.). Before inferential analyses, normality was assessed using the Shapiro-Wilk test. Because of the pilot nature of the study, the relatively small sample size, and the deviation of some outcome distributions from normality, nonparametric tests were applied. Specifically, we used a simple nonparametric comparison of pretest-to-posttest change scores instead of model-based adjustment.

For within-group comparisons (pretest vs. posttest within each group), the Wilcoxon signed-rank test was used. For between-group comparisons, change scores were computed (Δ=post−pre) and compared between the VR group and the mannequin group using the Mann-Whitney U test. Effect sizes for nonparametric tests were calculated as r=|Z|/√N, where Z is the standardized test statistic and N represents the number of observations (non-zero pairs for Wilcoxon signed-rank tests and total sample size for Mann-Whitney U tests). All analyses were 2-tailed with a significance level of 0.05, and descriptive and inferential statistics (e.g., mean, standard deviation, median, mean change, and P-values) were reported for each variable. Although nonparametric tests were applied because of non-normal distributions in some variables, mean and standard deviation are also presented for descriptive purposes to facilitate interpretation of the magnitude of change. The raw data supporting the findings of this study are available as Dataset 1.

## Results

### Feasibility outcomes

A total of 40 medical interns were enrolled during the study period. The recruitment rate could not be formally calculated because the total number of eligible interns invited was not systematically tracked; however, all 40 who expressed interest and met preliminary criteria were enrolled. Of these, 5 (12.5%) were excluded before randomization due to the following reasons: eye-tracking calibration failure (n=2; 5.0% technical failure rate), psychoactive medication use (n=1; 2.5%), recent sleep deprivation (n=1; 2.5%), and incomplete participation (n=1; 2.5%). Consequently, the randomization rate among enrolled participants was 87.5% (35 of 40). All 35 randomized participants completed their assigned intervention (three sessions each), yielding an intervention completion rate of 100% (n=35/35). All participants also completed the posttest assessment, resulting in a posttest completion rate of 100% (n=35/35). No additional technical failures were encountered during the VR sessions beyond the two initial calibration failures.

### Baseline characteristics

A total of 35 medical interns participated (VR group: n=17; mannequin group: n=18). The mean age was 26.12±2.03 years in the VR group and 26.67±2.11 years in the mannequin group. At baseline, the two groups did not differ significantly on SA (P=0.443) or error scores (P=0.782). For ATLS performance, the difference was not statistically significant (P=0.062); however, the mannequin group had numerically higher pretest scores than the VR group (mean±SD, 10.61±12.88 vs. 2.35±13.13), which should be taken into account when interpreting change scores (Table 1). In the VR group, the mean SA score increased from 6.53±2.07 at pretest to 13.12±3.92 at posttest, while in the mannequin group, SA increased from 7.56±3.37 to 10.67±3.69. SA improved significantly within both groups (VR: P<0.001, r=0.88; mannequin: P<0.001, r=0.88). Between-group comparison of change scores (Δ=post−pre) using the Mann-Whitney U test suggested a greater improvement in the VR group (P=0.006, r=0.46), though this exploratory finding should be viewed with caution (Table 2).

ATLS scores in the VR group increased from 2.35±13.13 to 24.47±15.17, while the mannequin group improved from 10.61±12.88 to 18.83±13.67. ATLS scores improved significantly after the intervention in both groups (VR: P=0.001, r=0.83; mannequin: P<0.001, r=0.88). Between-group comparison of change scores using the Mann-Whitney U test indicated a larger point estimate of improvement in the VR group (P=0.003, r=0.48) in this exploratory analysis (Table 2).

### Error

**Fig. 3.**
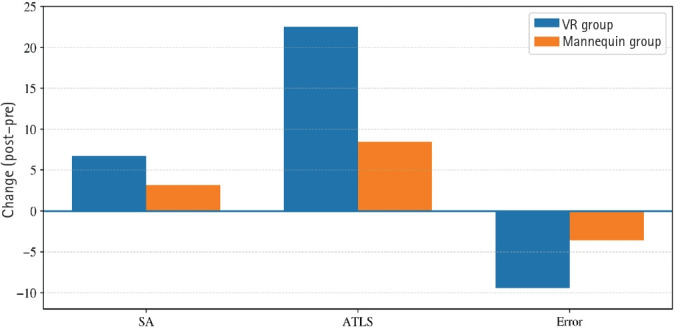
Comparison of situational awareness (SA), Advanced Trauma Life Support (ATLS), and error change scores between virtual reality (VR) and mannequin groups.

The VR group showed a reduction in error scores from 14.65±7.76 to 5.29±5.06, while in the mannequin group, errors decreased from 14.67±6.24 to 11.06±5.07. Analysis revealed a significant decrease in errors within both groups (VR: P=0.004, r=0.69; mannequin: P<0.001, r=0.88). Between-group analysis of change scores using the Mann-Whitney U test showed that the VR group showed a numerically greater reduction in errors (P=0.005, r=0.47), interpreted as a preliminary signal (Table 2, Fig. 3).

## Discussion

### Key results

As a pilot randomized controlled trial designed to assess feasibility and explore preliminary signals of potential benefit rather than provide definitive comparative effectiveness estimates, this study found that both simulation modalities eye-tracking–supported VR and mannequin-based simulation were associated with improvements in SA, ATLS performance, and trauma-management error scores from pretest to posttest. Although baseline ATLS scores did not differ significantly between the groups, the mannequin group exhibited numerically higher pretest scores. Nevertheless, the overall pattern of results indicated that the VR plus eye-tracking feedback arm achieved greater gains in SA, ATLS performance, and error reduction than the mannequin-based group.

### Interpretation

The improvements observed in both groups are consistent with the recognized benefits of simulation-based education, where repeated exposure to structured scenarios and targeted feedback may reinforce attention prioritization and rapid decision-making under pressure [4,5]. The pattern of numerically greater gains in the VR group could reflect several complementary features of the intervention: the use of gaze heatmaps to visualize attentional allocation may help learners recognize and address gaps in cue detection [8], while the immersive, interruption-rich VR environment may more closely approximate the cognitive demands of real ED settings. However, because the VR and mannequin conditions differed in multiple respects (e.g., platform, immersion, and feedback modality), the relative contribution of any single component including eye-tracking remains uncertain. These possibilities should therefore be regarded as tentative explanations requiring further investigation rather than as demonstrated mechanisms.

### Comparison with previous studies

The present findings align with prior work suggesting that simulation-based education can improve SA and clinical performance while reducing errors [4,5], and they extend the growing literature on integrating eye-tracking into simulation. O’Meara et al. [10] demonstrated that video-based eye-tracking feedback improved paramedicine and nursing students’ attention to critical cues, while Hanke et al. [11] reported distinct gaze profiles between less-experienced and expert clinicians in trauma VR. The current study adds to these observations by embedding eye-tracking-derived heatmaps directly into instructor-led debriefing within an interruption-rich immersive VR environment and by examining ATLS performance and error outcomes in medical interns. Consistent with Braund et al. [8], who noted the potential of eye-tracking-augmented debriefing, our results suggest that such an approach may be feasible and could offer preliminary signals of benefit, though all between-group comparisons remain exploratory.

Recent work further highlights the value of eye-tracking metrics for capturing attention allocation. Sugimoto et al. [12] used eye-tracking to assess situation awareness during nursing training, showing that visual attention patterns are associated with clinical decision-making. Qu et al. [13] demonstrated that machine-learning models applied to gaze data can predict an individual’s SA level, supporting the notion that eye-tracking provides a rich, objective window into cognitive processes underlying SA. In pediatric trauma simulation, Damji et al. [14] emphasized the safety importance of sustained patient-focused attention, a finding that resonates with the present study’s use of heatmaps to help interns identify attentional gaps. Together, these studies illustrate the multifaceted potential of eye-tracking for both assessment and feedback, while also indicating that there is no single universal gaze pattern for effective SA [15], a nuance that cautions against overinterpreting any single metric. The present pilot trial, therefore, adds to this body of work by offering preliminary evidence that eye-tracking-based feedback may complement simulation-based trauma training; however, these signals require confirmation in larger, controlled studies.

### Generalizability

Both eye-tracking–supported VR and mannequin-based simulation were associated with improvements in SA, ATLS performance, and reductions in trauma-management errors, suggesting that structured simulation with targeted feedback may be relevant for trauma training in ED-like settings that require rapid cue recognition, attention prioritization, and time-pressured decision-making. However, generalizability may be limited by the small, single-center convenience sample, potential modality-related differences in perceived realism and cognitive load, reliance on qualitative eye-tracking feedback (instructor-interpreted heatmaps rather than quantitative gaze metrics), the absence of long-term follow-up, and practical barriers such as calibration demands, equipment requirements, and the exclusion of learners with visual impairments.

### Suggestions for further studies

Future research could build on these preliminary findings by using larger, multicenter randomized designs across different learner levels and clinical roles. Studies may benefit from integrating quantitative eye-tracking metrics (e.g., fixation duration, scan paths, and dwell time on critical cues) within standardized feedback protocols and by comparing different feedback approaches (instructor-led vs. data-driven vs. self-review). Future work could also examine skill retention and transfer to clinical performance, explore hybrid models combining VR with hands-on skill components, and include feasibility and cost-effectiveness evaluations alongside measures of cognitive load, usability, and VR-related side effects.

### Conclusion

In this pilot study, both simulation modalities were associated with pre-to-post improvements in SA, ATLS performance, and error scores. The VR plus eye-tracking feedback arm showed numerically larger point estimates of change; however, as a pilot trial, these between-group differences are exploratory and should not be interpreted as confirmatory evidence of superiority. Larger, adequately powered studies are needed to evaluate effectiveness.

### Limitations

This study has several limitations that should be considered when interpreting the findings. First, the small sample size and single-center design limit the generalizability of the results, and the use of convenience sampling may increase the risk of selection bias. In addition, modality-related differences in perceived realism and cognitive load between the VR environment and mannequin-based training may have contributed to performance differences. From a technical standpoint, eye-tracking data were used qualitatively through instructor-interpreted heatmaps rather than through quantitative analyses of gaze coordinates, scan paths, or fixation durations; incorporating quantitative metrics could enable more standardized feedback and a more detailed assessment of attentional processes. The absence of long-term follow-up, limited assessment of hands-on physical skills, and potential variation in learners’ exposure to scenario repetition may also have affected the stability and consistency of outcomes. Finally, practical considerations, including calibration requirements, exclusion of learners with visual impairments, and reliance on specialized equipment, may constrain broader implementation.

## Figures and Tables

**Figure f4-jeehp-23-08:**
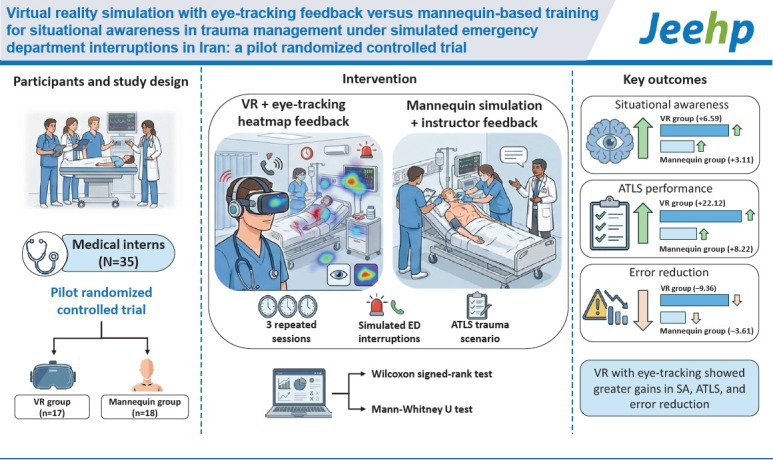


**Table 1.** Baseline characteristics and pretest performance by group

Variable	VR (n=17)	Mannequin (n=18)	P-value^a)^
Age (yr)	26.12±2.03	26.67±2.11	0.424
SA (pretest)	6.53±2.07	7.56±3.37	0.443
ATLS (pretest)	2.35±13.13	10.61±12.88	0.062
Errors (pretest)	14.65±7.76	14.67±6.24	0.782

Values are presented as mean±standard deviation. VR, virtual reality; SA, situational awareness; ATLS, Advanced Trauma Life Support.

a) P-values from the independent-samples Mann-Whitney U test (2-sided).

**Table 2.** Descriptive statistics and within- and between-group comparisons of SA, ATLS, and error scores

Outcome/group	Pretest mean±SD	Posttest mean±SD	Δ Change mean±SD	P-value (within-group sig.)^^a)^^	Within-group r^^a)^^	P-value (between-group sig.)^^b)^^	Between-group r^^b)^^	
SA						**0.006**	**0.46**	
VR	6.53±2.07	13.12±3.92	+6.59±4.03	<0.001	0.88			
Mannequin	7.56±3.37	10.67±3.69	+3.11±2.59	<0.001	0.88			
ATLS						**0.003**	**0.48**	
VR	2.35±13.13	24.47±15.17	+22.12±15.23	0.001	0.83			
Mannequin	10.61±12.88	18.83±13.67	+8.22±7.81	<0.001	0.88			
Error						**0.005**	**0.47**	
VR	14.65±7.76	5.29±5.06	−9.36±9.51	0.004	0.69			
Mannequin	14.67±6.24	11.06±5.07	−3.61±3.20	<0.001	0.88			

Effect size: r=|Z|/√N (Wilcoxon: N=non-zero pairs; Mann-Whitney: N=total sample). All tests were 2-tailed, α=0.05. Statistically significant results are marked in bold. SA, situational awareness; ATLS, Advanced Trauma Life Support; SD, standard deviation; VR, virtual reality.

^a)^By Wilcoxon signed-rank test (within-group). ^b)^By Mann-Whitney U test (between-group).

## Footnotes

**Data availability**
Data files are available from https://doi.org/10.7910/DVN/CJZC8H Dataset 1. Two-Group Dataset of Situational Awareness, ATLS Performance, and Error Score.

## Supplementary materials

Supplement 1. Virtual reality content scenario with eye-tracking.

Supplement 2. Modified SAGAT situational awareness assessment checklist.

Supplement 3. ATLS performance and error assessment checklist.

Supplement 4. Audio recording of the abstract.
