# Supplementary material for: Virtual reality simulation with eye-tracking feedback versus mannequin-based training for situational awareness in trauma management under simulated emergency department interruptions in Iran: a pilot randomized controlled trial
Source: J Educ Eval Health Prof. 2026 Apr 29;23:8. doi: 10.3352/jeehp.2026.23.8 (PMC13222740; doi:10.3352/jeehp.2026.23.8)
Supplement: Supplementary file 2 — Supplement 1. Virtual reality content scenario with eye-tracking. [file jeehp-23-08-suppl1.docx]

**Virtual Reality (VR) Content Scenario with Eye-Tracking**
**Topic:** Management of a Multiple-Trauma Patient (Polytrauma)
**Learner Role:** ATLS Team Leader (Emergency Physician)
**Total Duration:** 242 seconds

**Scenario Description**

It is a busy, high-stress day in the Emergency Trauma Center. The environment is saturated with the sound of sirens, crying, groaning, and continuous movement of nursing staff. Beds are rapidly filled, and the clinical workload places substantial pressure on the healthcare team.

On one of the stretchers lies a **28-year-old male** in **critical condition**. His face and head are covered in blood; his clothing is torn; and his **left lower limb has sustained traumatic amputation with ongoing active hemorrhage**. His breathing is harsh and accompanied by **audible stridor**, and blood intermittently appears at the corner of his mouth. On auscultation, the **left lung demonstrates markedly diminished/absent breath sounds**, and a **small open wound is present on the chest wall**.

The vital signs monitor displays **profound hypotension** (BP **69/38**), **severe tachycardia** (PR **178**), and **reduced oxygen saturation** (SpO₂ **87%**). A resident physician is standing at the bedside and, with visible anxiety, turns to you (the emergency physician) and asks:
**“Doctor, what should be the first intervention for this patient?”**
All eyes are on you. You must immediately announce life-saving decisions and provide clear direction for the team’s next steps.

However, the situation around the bed is highly chaotic and distracting:

- The patient’s mother is crying and agitated at the bedside, repeatedly calling for the physician.
- A companion of another ambulatory injured patient approaches while holding the patient’s arm and loudly demands priority:
  **“Doctor! Why is no one paying attention to us? My patient is bleeding too!”**
- Simultaneously, the family of a patient who died moments earlier move toward the current bedside. One confronts the physician:
  **“How did our patient die? Why didn’t you do anything?”**
  Their voices escalate and the confrontation intensifies.
- At the same time, a medical student disrupts the resident’s concentration with continuous, irrelevant questions.
- The suction device malfunctions; blood accumulates in the patient’s mouth, further complicating airway management.
- Amid the crowding, an emergency services worker bends down to clean spilled blood on the floor—directly under the emergency physician’s feet—while the physician attempts to control the situation.

The environment is saturated with anxiety, noise, and time pressure. Every second is critical, and your responses to the resident may represent the boundary between survival and death for this young patient.

**Key Interruption Points for Eye-Tracking**

**Intervals:** [S0–S66] | [S66–S101] | [S101–S156] | [S156–S242] | [S0–S242]

**Phase 1: S0 – S66**

A **28-year-old** trauma patient with injuries to the **head**, **chest**, and **traumatic limb amputation** is on the emergency stretcher. He has **depressed level of consciousness** (**GCS 7/15**), **active hemorrhage**, and **audible stridor**. The resident stands beside the bed, looks to you (the emergency physician), and asks:

**“Doctor, what should be the first step right now? I think we have three options: insert a chest tube, give IV fluids, or intubate.”**
⏱ **Response time:** 15 seconds

**Phase 2: S66 – S101**

The resident initiates **endotracheal intubation** and instructs the nurse to elevate the bed. The resident then asks:

**“Doctor, what is the appropriate tube size? Which type of tube should I choose, and how far should it be advanced into the trachea?”**
⏱ **Response time:** 15 seconds

**Phase 3: S101 – S156**

The resident attempts intubation but fails on the first attempt. The patient’s mouth is filled with blood. The resident orders suctioning. A nurse asks a student nurse to bring the suction machine. The device is connected, but it does not turn on. The nursing team becomes engaged in troubleshooting, yet suction remains unavailable. With worsening hypotension and oxygen desaturation, the resident looks at you with concern and asks:

**“Doctor, what should I do now? Try intubation again? Perform a cricothyroidotomy? Or place an LMA?”**
⏱ **Response time:** 15 seconds

**Phase 4: S156 – S242**

The resident proceeds with **cricothyroidotomy**. At this moment, a nurse loudly states:

**“Doctor! The patient has gone into shock!”**
All attention shifts to the monitor. The resident, visibly distressed, asks:

**“Doctor, what should I do now? Should I continue? Give an antiarrhythmic? Deliver a shock?”**
⏱ **Response time:** 15 seconds

Following intervention and defibrillation, the patient’s rhythm returns to normal. The cricothyroidotomy is completed, the patient is stabilized, and he is subsequently transferred to the **Intensive Care Unit (ICU)**.

**Phase 5: S0 – S242**

**Eye-tracking assessment** from the beginning to the end of the scenario content.
