# Supplementary material for: Virtual reality simulation with eye-tracking feedback versus mannequin-based training for situational awareness in trauma management under simulated emergency department interruptions in Iran: a pilot randomized controlled trial
Source: J Educ Eval Health Prof. 2026 Apr 29;23:8. doi: 10.3352/jeehp.2026.23.8 (PMC13222740; doi:10.3352/jeehp.2026.23.8)
Supplement: Supplementary file 3 — Supplement 2. Modified SAGAT situational awareness assessment checklist. [file jeehp-23-08-suppl2.docx]

**Modified SAGAT Situational Awareness Assessment Checklist**

Scenario:

A 28-year-old male with multiple traumatic injuries from a motor-vehicle collision is lying on the bed. His level of consciousness is GCS: 7/15. He has head trauma and a chest injury, a traumatic amputation of a lower limb, and active hemorrhage. Blood is visible on the patient’s face and torn clothing. The patient’s left leg is amputated and has active bleeding. The conjunctiva are pale. He has stridorous breath sounds, and a small amount of blood is coming from the corner of his mouth. Breath sounds on the left are decreased, and there is a 2‑cm wound on the chest.

BP: 69/38 PR: 178 SpO2: 87% RR: 37

How to complete the questionnaire:

The SAGAT method, based on the use of a simulator, works as follows: at specified intervals, the researcher pauses the simulation and asks the participant researcher-developed questions. These questions are designed according to the objectives of the educational intervention and the job/task analysis. After the participant answers, the responses are evaluated and—after being matched to the simulation state—are categorized as correct or incorrect. Then, in line with the intervention objectives, the participant’s situation awareness is measured. The questions are asked at three levels of Endsley’s hierarchical theory of situation awareness, which includes three freezes during the simulation to assess situation awareness at each of the following levels:

1. Perception of the situation: What has happened?

2. Comprehension/interpretation of the situation and the meaning of the information: What does this mean?

3. Projection of future status and correct decision-making (What consequences will it have for the patient?)

The freezes should be scheduled randomly throughout the simulation.

At least 3–5 minutes after the start of the trial, perform the first freeze.

There should be at least 1 minute between two simulation freezes.

**Modified SAGAT Situational Awareness Assessment Checklist**

| Questions | Answer | Correct or Incorrect (tick) |
| --- | --- | --- |
| Perception of the situation: What has happened? | | |
| What is the patient’s respiratory status? (lung auscultation) |  |  |
| What is the current SpO2? |  |  |
| What is the patient’s heart rate? |  |  |
| Approximately how much blood has the patient lost? |  |  |
| Are there obvious signs of external bleeding? |  |  |
| How much bleeding is coming from the amputated limb? |  |  |
| Comprehension/interpretation and meaning of the information: What does this mean? | | |
| Does the patient require immediate intervention for airway, breathing, or circulation? |  |  |
| What is the first action that should be performed for this patient? |  |  |
| For intubation, what tube size would you choose, and to what depth should it be advanced into the trachea? |  |  |
| Does the patient need an immediate blood transfusion? |  |  |
| If intubation is not possible due to jaw/facial injuries and suction is ineffective, what is the next action for the patient? |  |  |
| If the patient develops shock during the initial incision step of a cricothyroidotomy, what should be done next? |  |  |
| Projection and correct decision-making (What consequences will it have for the patient?) | | |
| In the next minute, do you expect the patient’s blood pressure to increase, decrease, or remain unchanged? |  |  |
| Do you expect any change in the patient’s heart rate in the next minute? |  |  |
| How long do you think it will take for the patient’s oxygen saturation to return to normal? |  |  |
| Will the patient require an immediate blood transfusion in the unit/ward? |  |  |
| After ED management, which unit do you think the patient will be transferred to? |  |  |
| How will the patient’s injuries affect his long-term health? |  |  |
| What is the patient’s chance of survival? |  |  |
| Total number of correct answers | |  |
