# Supplementary material for: Virtual reality simulation with eye-tracking feedback versus mannequin-based training for situational awareness in trauma management under simulated emergency department interruptions in Iran: a pilot randomized controlled trial
Source: J Educ Eval Health Prof. 2026 Apr 29;23:8. doi: 10.3352/jeehp.2026.23.8 (PMC13222740; doi:10.3352/jeehp.2026.23.8)
Supplement: Supplementary file 4 — Supplement 3. ATLS performance and error assessment checklist. [file jeehp-23-08-suppl3.docx]

**ATLS Performance and Error Assessment Checklist**

Scenario:

A 28-year-old male with multiple traumatic injuries from a motor-vehicle collision is lying on the bed. His level of consciousness is GCS: 7/15. He has head trauma and a chest injury, a traumatic amputation of a lower limb, and active hemorrhage. Blood is visible on the patient’s face and torn clothing. The patient’s left leg is amputated and has active bleeding. The conjunctiva are pale. He has stridorous breath sounds, and a small amount of blood is coming from the corner of his mouth. Breath sounds on the left are decreased, and there is a 2‑cm wound on the chest.

BP: 69/38

PR: 178

SpO2: 87%

RR: 37

*Questions are presented in order: the equipment on the table corresponds to the required interventions (the student, in a dynamic environment with interruption factors, must choose the interventions). Simultaneously, a 360-degree video recording of the student’s interaction will be performed. (Each station: 5 minutes)

Checklist

| Action priority | Score and error criteria | Tick | Interruption occurring | Decision-making time |
| --- | --- | --- | --- | --- |
| Selection of the first action for the casualty: | | |  |  |
| Endotracheal intubation | 5 |  |  |  |
| Insert chest tube | 4 |  |  |  |
| IV fluid therapy | 3 |  |  |  |
| Apply pneumatic tourniquet cuff | 2 |  |  |  |
| Send to radiology | 4-(error recorded) |  |  |  |
| None (action not listed) | 4-(error recorded) |  |  |  |
| If intubation is selected: (Which tube is chosen?) | | |  |  |
| ET tube 8.0 | 5 |  |  |  |
| ET tube 6.0 | 1 |  |  |  |
| ET tube 5.0 | 0(error recorded) |  |  |  |
| None | 0(error recorded) |  |  |  |
| If a tube is selected (To what depth is it advanced into the trachea?) | | |  |  |
| 23 cm | 5 |  |  |  |
| 21 cm | 1 |  |  |  |
| 18 cm | 0(error recorded) |  |  |  |
| None (out of range) | 0(error recorded) |  |  |  |
| Next actions: If chest tube insertion is selected: Which tube is chosen? | | |  |  |
| 28 Fr tube | 5 |  |  |  |
| 24 Fr tube | 1 |  |  |  |
| 40 Fr tube | 5- (error recorded) |  |  |  |
| None | 5- (error recorded) |  |  |  |
| After selecting the tube: How far should it be advanced? | | |  |  |
| To 10 cm beyond the last side-hole mark | 5 |  |  |  |
| At the level of the last side-hole mark | 1 |  |  |  |
| To 20 cm beyond the last side-hole mark | 5- (error recorded) |  |  |  |
| None | 5- (error recorded) |  |  |  |
| Next actions: If IV fluid therapy is selected | | |  |  |
| 250 mL and repeat up to 8 times | 5 |  |  |  |
| 2 L rapid bolus | 5- (error recorded) |  |  |  |
| 1 L per hour | 5- (error recorded) |  |  |  |
| None | 5- (error recorded) |  |  |  |
| If IV fluid therapy is selected How should blood pressure changes be managed? | | |  |  |
| Systolic blood pressure should not exceed 90 mmHg | 5 |  |  |  |
| Raise blood pressure to 110/70 | 5- (error recorded) |  |  |  |
| Raise blood pressure to 120/80 | 5- (error recorded) |  |  |  |
| None | 5- (error recorded) |  |  |  |
| Next actions: If a pneumatic tourniquet is selected To what pressure should it be inflated? | | |  |  |
| To 100 mmHg | 5 |  |  |  |
| To 160 mmHg | 3- (error recorded) |  |  |  |
| To 60 mmHg | 3- (error recorded) |  |  |  |
| None | 3- (error recorded) |  |  |  |
| If the tourniquet is selected For how long should it be set? | | |  |  |
| Maximum 2 hours | 5 |  |  |  |
| Maximum 6 hours | 3- (error recorded) |  |  |  |
| Maximum 1 hour | 3- (error recorded) |  |  |  |
| None | 3- (error recorded) |  |  |  |
| Next actions: If radiology is selected and a tension pneumothorax is seen on the X-ray, what is the next action? | | |  |  |
| Insert chest tube | 5 |  |  |  |
| Needle decompression of the chest | 1 |  |  |  |
| Asherman chest seal (occlusive dressing) | 0(error recorded) |  |  |  |
| None | 0(error recorded) |  |  |  |
| Final step: Airway was secured with intubation. A CHEST TUBE was inserted; the patient’s blood pressure and level of consciousness improved. BP 110/67 PR 100 GCS 13/15 Two liters of blood collected in the chest drainage bottle. What is the next action? | | |  |  |
| General surgery operating room | 4 |  |  |  |
| Transfuse 2 units of blood | 2 |  |  |  |
| Chest CT scan | 2-(error recorded) |  |  |  |
| Emergency thoracotomy | 4-error recorded) |  |  |  |
| Total score | | | |  |
| Total errors | | | |  |
